# Long-term culture of cholangiocytes from liver fibro-granulomatous lesions

**DOI:** 10.1186/1471-230X-6-13

**Published:** 2006-04-03

**Authors:** Luciana B Chiarini, Christina M Takiya, Radovan Borojevic, Alvaro NA Monteiro

**Affiliations:** 1 Departamento de Bioquímica, Instituto de Química, Universidade Federal do Rio de Janeiro, Rio de Janeiro 21949, Brazil; 2 Departamento de Histologia e Embriologia, Instituto de Ciências Biomédicas, Universidade Federal do Rio de Janeiro, Rio de Janeiro 21941, Brazil

## Abstract

**Background:**

Extensive bile duct proliferation is a key feature of the tissue reaction to clinical and experimental forms of liver injury. Experimental infection of mice by *Schistosoma mansoni *is a well-studied model of liver fibrosis with bile duct hyperplasia. However, the regulatory mechanisms of bile duct changes are not well understood. In this study we report the reproducible isolation of long-term cultures of cholangiocytes from mice livers with schistosomal fibrosis.

**Methods:**

We have isolated a cholangiocyte cell line from *Schistosoma*-induced liver granulomas using a combination of methods including selective adhesion and isopyknic centrifugation in Percoll.

**Results:**

The cell line was characterized by morphological criteria in optical and transmission electron microscopy, ability to form well differentiated ductular structures in collagen gels and by a positive staining for cytokeratin 18 and cytokeratin 19. To our knowledge, this is the first murine cholangiocyte cell line isolated from schistosomal fibrosis reported in the literature.

**Conclusion:**

After 9 months and 16 passages this diploid cell line maintained differentiated characteristics and a high proliferative capacity. We believe the method described here may be a valuable tool to study bile duct changes during hepatic injury.

## Background

**Figure 1:**
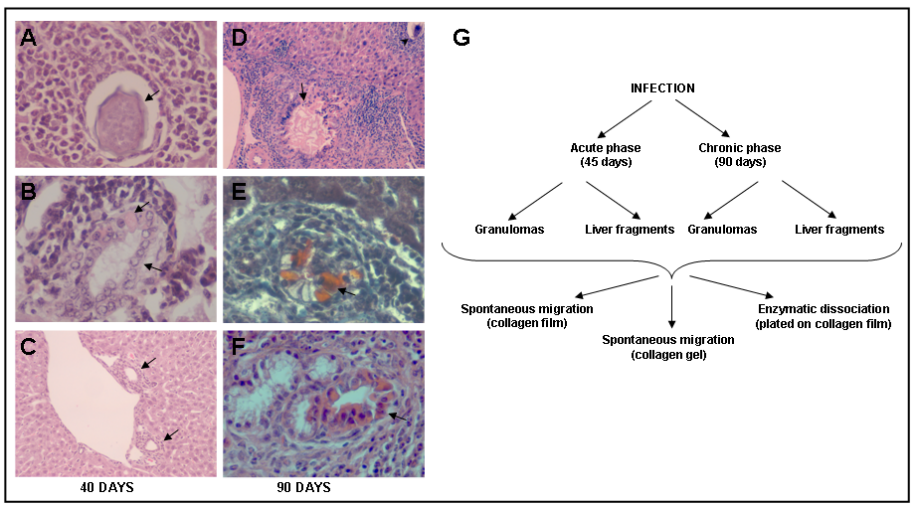
**Schistosomal granulomas and outline of method used to isolate bile ductal cell lines**. **A**. Schistosomal egg (arrow) in a portal vein with initial granuloma formation after 40 days of infection (acute phase). Portal space with mieloid progenitors. Hematoxylin-Eosin staining (400×). **B**. Portal space with a bile duct hyperplasia (arrows) with clear cells after 40 days of infection (acute phase). Hematoxylin-Eosin staining (400×). **C**. Portal space with biliary ducts (arrows) after 40 days of infection (acute phase). Hematoxylin-Eosin staining (100×). **D**. Enlarged inflammed portal space containing a hyperplastic bile duct with cristal-like structures (arrow) after 90 days of infection (chronic phase). Note also a Schistosomal egg granuloma (arrowhead). Hematoxylin-Eosin staining (100×). **E**. Enlarged inflammed portal space containing a hyperplastic bile duct with mucinous metaplasia (arrow) after 90 days of infection (chronic phase). Masson's trichrome staining (400×). **F**. Hyperplastic bile ducts with mucinous metaplasia (arrow) surrounded by eosinophyls and myeloid progenitors after 90 days of infection (chronic phase). Hematoxylin-Eosin staining (400×).**G**. Diagram outlining the different sources used to isolate bile ductal cell lines.

Extensive bile duct proliferation is a key feature of the tissue reaction to clinical and experimental forms of liver injury and in many cases, this proliferation may affect liver function [1,2]. It has long been appreciated that bile duct epithelial cells can be isolated and cultured *in vitro *from human [3-7] and animal liver tissue [8-12]. Cultures of bile duct epithelia have been derived from normal, cholestatic, or carcinogen-treated livers [10,11]. Although experimental infection of mice by *Schistosoma mansoni *is a well studied model of liver fibrosis with bile duct hyperplasia [13](Fig. 1A–F), cholangiocytes have not yet been isolated from schistosomal livers and characterized *in vitro*. In experimental schistosomiasis, a spectrum of pathologic changes of the intrahepatic bile ducts can be observed, such as hyperplasic epithelial lining made up of hypertrophic cells or cells with nuclei disposed in variable height and periductular fibrosis [13]. The origin and nature of these bile duct cells remain unknown since there has been no systematic study of the cells implicated in bile duct hyperplasia during *Schistosoma *infection.

Since certain chronic disorders of the biliary tract have biliary epithelial cells as their primary targets [11,14,15], the ability to culture these cells would be important to study other diseases. Moreover, cell lines isolated from human or experimental sources show a restricted capacity of proliferation *in vitro*. Alternatively, cell lines derived from carcinogen-treated animals have extended proliferative capacity but do not display differentiated characteristics [11].

In the present study we have developed a method to isolate a bile duct cell line from schistosomal liver granulomas (shown in Fig. 1A–G) by isopyknic centrifugation on Percoll and selective adhesion. This cell line displays extended proliferative capacity while maintaining differentiated characteristics. We also describe an in vitro model to approach ductular morphogenesis using collagen gels.

## Methods

### Formation and isolation of granulomas or liver fragments

C3H/HeN mice of both sexes were infected by transcutaneous penetration of 40 cercariae of *Schistosoma mansoni *(BH strain, Instituto Oswaldo Cruz, Rio de Janeiro). Mice were sacrificed after 45 (acute phase) or 90 (chronic phase) days of infection. At this time livers were either cut in 1 mm^3 ^fragments or granulomas were isolated from liver tissue by homogenization and sedimentation [16]. Mice were obtained from the Institute of Chemistry mouse breeding facility, and used following the authorization of the Institutional Committee for Research and Animal Studies. The animals were handled following the Federal University of Rio de Janeiro guidelines for the use ofexperimental animals.

### Cell cultures

We initiated cultures from granulomas or liver fragments from chronic or acute phase schistosomal livers (Fig. 1A–F) by three different approaches (Fig. 1G): **a**) *Spontaneous migration of cells onto a type I collagen film*. Granulomas were plated in 25 cm^2 ^tissue culture flasks with enough medium just to cover the granulomas in order to avoid floating. Liver fragments were plated 12 fragments per plate covered by a small drop of medium, allowed to adhere and warm medium was added after 2 h. Cultures were maintained in the Dulbecco's Modified Minimum Essential Medium (DMEM) with 10% fetal bovine serum (FBS – Cultilab, Campinas, Brazil) under a humidified atmosphere with 5% CO_2_. These conditions were used for all the cultures throughout this study; **b**) *Collagen gel*. Liver fragments or granulomas were embedded in a gelling solution of type I collagen gel; **c**) *Enzymatic dissociation*. After isolation, granulomas were washed twice with balanced saline solution (BSS) for 15 min each at 37°C with a magnetic stirrer. BSS was then changed for a 1 mg/ml collagenase solution type IA (Sigma Chemical Company, St Louis, MO) in DMEM pH 7.4 and incubated four times for 1 h each at 37°C with magnetic stirrer. After each collagenase treatment cells were collected and centrifuged in a clinical centrifuge, washed twice in BSS and plated. After 24 h the culture was washed and medium changed. Liver fragments were first incubated in 0.125% trypsin (Sigma) in calcium and magnesium-free BSS, pH 7.8 on ice for 30 min to allow trypsin to penetrate the tissue. Subsequently, trypsin was allowed to act by incubation at 37°C with a magnetic stirrer. Trypsin was then taken out and tissue fragments were subjected to the same treatment described for isolated granulomas.

### Extracellular matrix

Collagen type I was derived from rat tail tendon [17]. Collagen solution had a concentration range from 1 to 2 mg/ml prior to use. This solution was used directly for preparing gels and diluted to 0.3 mg/ml for films. Collagen IV was a commercially available preparation from Nikka (Japan). Collagen Type IV films were also made from a 0.3 mg/ml solution. Films were prepared by laying the collagen solution onto the plate and removing the excess fluid. Collagen gels were prepared in 60 mm plastic dishes by rapidly mixing 4.0 ml collagen solution, 1.0 ml DMEM (5X concentrated) and by raising the pH with 40 μl 1.0 N NaOH. When cells, granulomas or tissue fragments were to be plated onto the gel, the gel solution was dispensed on the plate, and after gel polymerization, it was rinsed three times with DMEM before plating. Alternatively, when the cells, granulomas or tissue fragments were to be included into the gel, they were quickly added to gel solution before the gel polymerization, and then the gel solution was dispensed on the plate.

### Percoll gradients

Isotonic Percoll solution (IPS) was made with 10X phosphate buffered saline (PBS) pH 7.4 and Percoll (Pharmacia, Uppsala, Sweden) (9:1 v/v). The IPS was diluted in DMEM containing 1% FBS. Pre-formed Percoll gradients were made by stepwise addition of 40, 50 and 60% IPS in the centrifuge tube. The cell suspension (1 × 10^7 ^cells/ml) was slowly placed on top of the gradient and centrifuged for 20 min at 1500 rpm in a refrigerated centrifuge at 22°C. Then, each fraction was collected, washed three times in BSS and plated in 60 mm tissue culture dishes coated with type I collagen film.

### Transmission electron microscopy

Whole gels were fixed with two incubations (10 and 50 min) of 4% glutaraldehyde/0.2 M cacodylate buffer (v/v). Gels were than rinsed three times for 10 min and once for 30 min with 0.4 M sucrose/0.2 M cacodylate buffer. Gels were post-fixed with two incubations (10 and 50 min) with 2% OsO_4 _/0.3 M cacodylate buffer and rapidly rinsed in distilled water. Dehydration was carried out through a graded series of ethanol 30, 50, 70, 95% each for 5 min and 100% twice for 20 min. Subsequently gels were further dehydrated with acetone twice for 15 min. Impregnation was done under vacuum for 4 h in Epon/acetone (1:2), and 4 h in Epon/acetone (1:1). Gels were incubated overnight in Epon and embedded in Epon with 1.7% DMP30. Semithin sections were stained with methylene blue and ultrathin sections were contrasted for 15 min with 7.5% uranyl acetate in ethanol, followed by 10 min in Reynold's lead citrate.

### Optical microscopy

Gels were fixed in neutral formalin and embedded in paraffin. Slides were stained using standard Groat's Hematoxylin counterstained with eosin. The Prenant's version of Masson's trichromic staining was used, with Harris' Hematoxylin, Rouge Ponceau/Orangé-G and Sulfo-green counter stains.

### Immunofluorescence

Immunofluorescence was performed on type I collagen-coated Lab-Tek 8 well culture slides (Miles Scientific, Naperville, IL). Cells were plated 1 × 10^4 ^per well and incubated for 48 h. Cells were subsequently washed three times with BSS and fixed and permeabilized with methanol for 4 min. Wells were washed with PBS for 5 min and incubated with 0.1% BSA in PBS to block unspecific binding. Cells were then briefly washed with PBS for 5 min and incubated with monoclonal antibodies against mouse cytokeratin-19, cytokeratin-18 (Amersham Laboratories, UK; gift from Dr. Wilson Savino) [18] or negative controls (an irrelevant mouse anti-rat IgG serum or no primary antibody) in 0.1% BSA in PBS different wells for 1 h at 37°C in a water bath. Cells were then washed three times with PBS and incubated with anti-IgG FITC conjugate (Amersham, UK) at 1:1000 dilution for 1 h at 37°C in the dark.

### Flow Cytometry analysis

Mixed and enriched cultures were trypsinized and single cell suspensions were made in DMEM. Flow cytometry analysis using forward-angle scatter as an indication of cell size was performed. Analysis was performed on a Counter's EPICS 751 flow cytometer.

### Karyotype

Karyotype analysis was performed by solid Giemsa staining of metaphase spreads as described [19].

## Results

### Primary cultures on collagen gels

All primary cultures in collagen gels irrespective of the approach used (whole or dissociated granulomas, whole or dissociated liver fragments) displayed extensive connective tissue outgrowth after ten days precluding any analysis of epithelial cells.

### Primary cultures on collagen films

**Figure 2:**
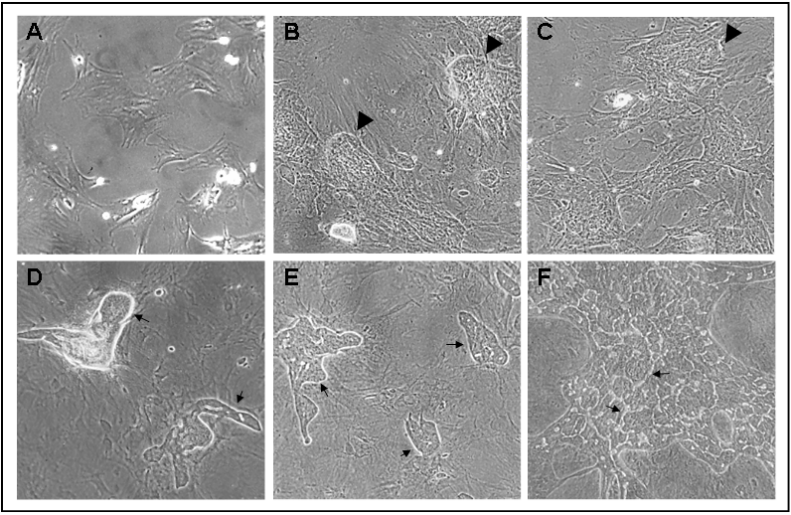
**Phase-contrast microscopy of cells derived from dissociated acute phase granulomas**. **A**. Homogeneous culture of liver connective tissue cells. **B**. Confluent stationary cultures presenting a monolayer of connective tissue cells and epithelial colonies (arrowheads). **C**. Patches of epithelioid morphology (arrowhead). **D **and **E**. Progressively differentiated epithelioid patches distinguished by light birefringence (arrows). **F**. Large epithelial patch with a network of small duct-like passages between cells (arrows). (750×).

Chronic and acute phase granulomas from liver produced small lymphocytes in the beginning of cultivation. Their migration diminished after the second and third days of culture and stopped at the end of the first week. Macrophages, recognized by their morphology, migratory behavior and their trypsin resistance, migrated from granulomas after the first day of culture. They proliferated intensely and formed distinct colonies. Acute phase granulomas in the first weeks of culture produced predominantly macrophages with progressive proliferation of connective tissue cells. Chronic phase granulomas showed a slower proliferation rate and produced predominantly connective tissue cells. At the end of the first month macrophage proliferation ceased. Small foci of epithelial cells were found in every culture. If cultures were trypsinized just after reaching confluence, homogeneous cultures of liver connective tissue cells were obtained (Fig. 2A). Alternatively most cultures were maintained as stationary cultures for more than 30 days after confluence. In these conditions, epithelial colonies also remained stationary and did not show extensive proliferation (Fig. 2B; arrowheads). At this stage, all cultures were subjected to weak trypsinization (no mechanical agitation). After this procedure, the cultures remained with some connective tissue cells and some epithelial foci. The remaining cells were kept and allowed to grow in standard conditions. Only cultures derived from dissociated acute phase granulomas showed extensive epithelial proliferation (described bellow). In all other cultures epithelial foci remained stationary and cultures were eventually dominated by fibroblastic outgrowth. All subsequent description refers to epithelial cells derived from dissociated acute phase granulomas.

### Formation of epithelial aggregates in primary cultures

Five to ten days after confluence, cultures showed distinct patches of epithelioid morphology (Fig. 2C; arrowheads). These patches seemed to differentiate progressively as judged by the appearance of a characteristic epithelial aspect with cells showing cobblestone morphology and a high nucleo/cytoplasmic ratio. They had usually 1 to 2 nucleoli and a more pronounced granularity. These patches could be distinguished from the connective tissue monolayer by a light birefringence on the border of the patches (Fig. 2D,E; arrows). Progressively, small duct-like spaces were formed between the cells, eventually coalescing in a complex array of passages (Fig. 2F; arrows). Lacking a three-dimensional scaffold, these patches formed large aggregates attached to the connective tissue stroma. Aggregates eventually detached from the monolayer and degenerated.

### Epithelial cells in collagen gels

**Figure 3:**
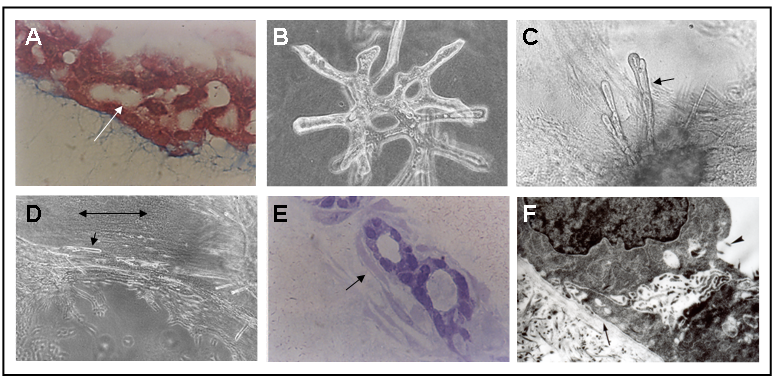
**Lumen formation**. **A**. Epithelial cell in heterogeneous culture plated on top of collagen gel. Section of paraffin-embedded gel stained with hematoxylin eosin (1250×). **B**. Branching epithelial organoid with lumen formation in collagen gel. Phase-contrast microscopy (250×). **C**. Branching epithelial organoid in collagen gel showing unistratified epithelium with lumen formation (arrow). Phase-contrast microscopy (250×). **D**. Mixed cell aggregates (connective and epithelial cells) in collagen gel. The double-headed arrow shows the direction of the preferential distortion of the matrix with alignment of collagen fibers between two aggregates. Note newly formed epithelial ducts following the direction of the aligned fibers. Phase-contrast microscopy (125×). **E**. Epithelial tubules in collagen gel. Semithin section of Epon-embedded gel, stained with methylene blue. Note unistratified epithelium, mucous-like substance in the lumen and close apposition of connective tissue cells (arrow) (940×). **F**. Epithelial tubules in collagen gel seen through electron microscopy. Note unistratified epithelium with microvilli protruding into the lumen (arrowhead) and a thin layer of basement membrane (arrow) (13500×).

A mixed population of epithelial and connective tissue cells was plated onto and into the collagen gels. When plated on the surface of the gel, epithelial cells grew as a monolayer but also showed multiple cavities inside and under the monolayer (Fig. 3A; arrow). When plated into the gel, isolated epithelial cells proliferated and formed three-dimensional organoids (Fig. 3B). These organoids grew in the multiple directions with extensive branching. Epithelial cells in the organoids had a high nucleo/cytoplasmic ratio and lumen formation was observed from the second day on (Fig. 3C; arrow). When mixed cell aggregates (connective tissue and epithelial cells) were included in the gel connective tissue migration resulted in the preferential distortion of the matrix with the alignment of collagen fibers between two aggregates [25](Fig. 3D; double-headed arrow). In these cases, newly formed epithelial ducts followed the direction of the aligned fibers (Fig. 3D; arrow).

In semithin sections connective tissue cells were seen in close apposition to the epithelial lining (Fig. 3E; arrows). In ultrathin sections, cells had an epithelial morphology and displayed long fine intermingled microvilli on lateral membranes between neighboring cells, as well as short microvilli protruding into the lumen (Fig. 3F; arrowhead). A thin basement membrane could (Fig. 3F; arrow) be seen together with a subjacent layer of fibrillar collagen. This layer of fibrillar collagen was likely synthesized *de novo *because the dimensions of the fibrils differed from the rat tail tendon collagen used to form the gel.

### Epithelial enrichment by selective adhesion

**Figure 4:**
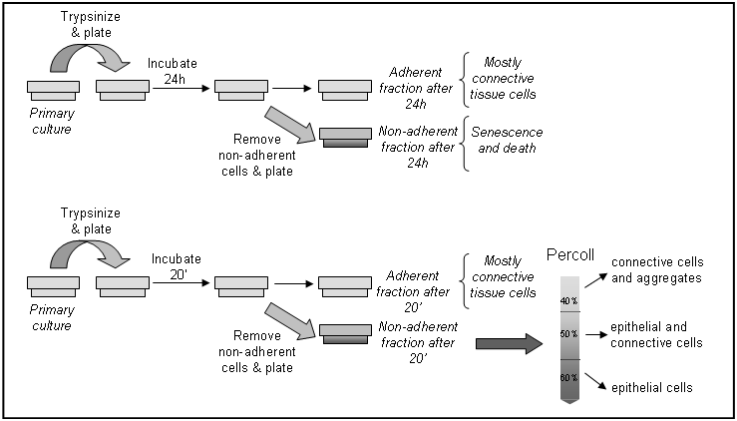
**Diagram outlining the strategy used to isolate bile ductal cell lines combining selective adhesion and Percoll gradient centrifugation**. Plates with dark bottom represent coating with collagen type IV film.

Monolayers containing epithelial cells were trypsinized and plated onto plastic (no collagen coating) flasks and incubated for 24 h. Then, the supernatant containing cells that did not attach to the substrate was collected and plated onto a flask covered with a collagen IV film. The first plating (adherent cells in 24 h) contained predominantly connective tissue cells and a few epithelial cells. In this flask, epithelial cells grew and differentiated forming aggregates. The second plating contained predominantly epithelial cells and a few connective tissue cells but only a small percentage of the cells remained viable after 24 h in the supernatant. In this case, epithelial cells in low density seemed to proliferate in an undifferentiated, highly spread morphology with low nucleo/cytoplasmic ratio and a clear cytoplasm. They eventually senesced and died (not shown). We repeated the experiment but instead of allowing 24 h for the first attachment we only allowed 20 min (Fig. 4). The first plating was similar to the one described above and contained predominantly connective tissue cells. The second plating contained a majority of epithelial cells and a considerable number of connective tissue cells. In this case, epithelial cells would proliferate in characteristic epithelial sheets. Cells derived from the non-adherent fraction after 20 min were used to isolate the epithelial cell line. Collagen type I and IV yielded similar results and in further experiments only collagen type I was used for coating the dishes.

### Percoll separation

Cells derived from non-adherent fraction (20-min procedure) were plated on a preformed Percoll gradient with three layers containing 40%, 50% and 60% of IPS. The fractions were collected and plated onto tissue culture flasks covered with collagen type I film to increase plating efficiency. The 60% fraction represented the epithelial cell enriched population. The 50% fraction contained epithelial cells but contaminating connective tissue was also present. The 40% fraction contained most of the connective tissue cells and large aggregates of non-dissociated epithelial cells.

### FACS analysis and immunofluorescence

**Figure 5:**
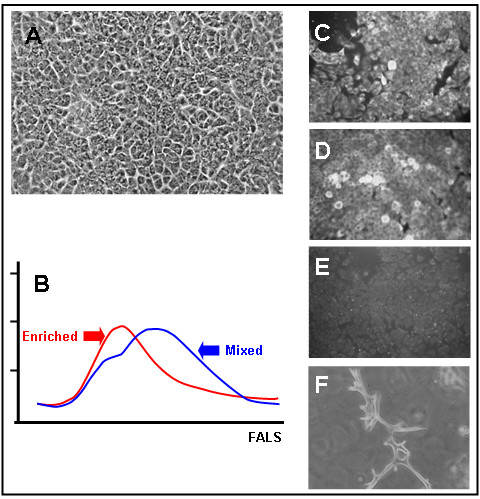
**Homogeneous cultures of ductal epithelial cells**. **A**. Phase contrast showing a homogeneous culture of epithelial cells. (750×). **B**. Comparative flow cytometry analysis of epithelial-enriched (red line) and mixed populations (blue line). X axis, Forward-angle light scatter representing cell size; Y axis, relative cell number. **C**. Immunofluorescence for cytokeratin-18 (600×). **D**. Immunofluorescence for cytokeratin-19 (600×) **E**. Negative control for immunofluorescence incubated with an irrelevant antibody (600×).**F**. Tubules of isolated cultures. Single-cell suspensions were allowed to proliferate. A previous layer of gel was made containing liver connective tissue cells as a feeder layer. Phase contrast microscopy (125×).

After Percoll separation and three further rounds of selective adhesion (20-min procedure), no more connective tissue cells adherent to the plastic surface during the incubation were found, and a morphologically homogenous epithelial cell line was obtained (Fig. 5A). A pool of cultures was made at this point. This cell line was analyzed by flow cytometry to determine distribution of cell sizes in the population. The enriched cell line and the mixed culture that had not been submitted to Percoll and selective adhesion procedure were analyzed (Fig. 5B). The two populations showed different patterns in relation to cell size. The heterogeneous population showed a peak of larger cells corresponding to connective tissue cells and a shoulder of smaller cells corresponding to the epithelial cells.

To further characterize the cell line we tested for epithelial markers cytokeratin-18 (present in hepatocytes and cholangiocytes) and cytokeratin-19 (present in cholangiocytes). The cell line was positive for both markers in nearly 100% of the cells (Fig. 5C,D).

### Tubules of isolated cultures

We included a single cell suspension of the isolated cell line inside a collagen gel. A previous layer of the collagen gel was made containing connective tissue cells to serve as a feeder layer. The epithelial cells proliferated and still maintained the capacity to form organoids in the gel (Figure 5E).

### Karyology

The number of chromosomes present in the epithelial cell line was counted after 16 and 18 passages. On both occasions the cell line displayed a normal diploid distribution with a modal number of 40 (not shown).

## Discussion

In the present study we established a long-term culture of cholangiocytes and described their behavior when embedded in a three-dimensional collagen matrix. We used a combined method of selective adhesion and Percoll density gradients to isolate this epithelial cell line.

During obstructive diseases, the intrahepatic biliary tree shows a marked structural modification including newly formed ductules originating from pre-existing ones with no junctions with the hepatic laminae [20]. On the other hand, in non-obstructive hepatic diseases newly formed ductules merge with the hepatic laminae and often end blindly in the fibrous septa [20].

The stimulation for bile duct cell proliferation may be regarded as intraluminal or interstitial. Intraluminal stimulation is present during chronic biliary tract obstruction in humans and after ligation of the common bile duct in experimental animals [21,22]. Interstitial stimulation is that associated with non-obstructive liver fibrosis. The latter is thought to be an epiphenomenon of hepatocyte necrosis [20]. However, fibrosis caused by *Schistosoma mansoni *infection presents extensive ductular hyperplasia without significant parenchymal injury [13]. Both fully differentiated hepatocytes and cholangiocytes can proliferate *in situ *in response to injury. Chronic cell injury such as chronic hepatitis elicit mobilization of hepatic endodermal stem cells or oval cells, resident in the Hering's canals, which can give rise both to hepatocytes and cholangiocytes [23]. Hyperplasia of oval cells has not been described in schistosomiasis, in accordance with absence of chronic parenchymal injury, and it is not probable that the described established cholangiocyte cell line derives from oval cells. This is also in accordance with its fully differentiated phenotype, growth in epithelial morphology, formation of duct-like structures, secretion of a basement membrane, as well as expression of the cytokeratins 18 and 19. Although these cells were derived from livers with gross morphological changes, it is noteworthy that in all three-dimensional cultures examined they formed a unistratified epithelium with lumen formation. Crystalloid elongated bodies and vacuolated or acidophilic cytoplasm, characteristic of hyperplasic bile ductal cells in schistosomiasis were not seen [13]. In a three-dimensional matrix, the cell line still retained the capacity to form organoid and subsequent branching without the need of direct contact of liver connective tissue cells.

Schistosomal granulomas are a rich source of cytokines and growth factors, whose role in connective tissue proliferation and activation as well as in fibroplasia is well established [24]. Extensive proliferation of inflammatory cells of myeloid origin associated with schistosomal granulomas is also well known, being limited to the perigranulomatous area [25]. In contrast, ductular hyperplasia in schistosomiasis is extensive and diffuse, associated with periductular fibrosis, and it is not topographically related to granulomas [13]. The role of cytokines produced by hepatic stellate cells and activated myofibroblasts in stimulation of cholangiocyte proliferation has been reported [26]. We propose that cholangiocyte hyperplasia is maintained in schistosomal liver by activated connective tissue, generating a proliferative and fully differentiated cell population. In the establishment of the described cholangiocyte cell line, this interaction was maintained in the beginning of cell cultures. The required condition for the final establishment of cholangiocyte cell lines may be separation of growing epithelial cells from the connective tissue, avoiding its overgrowth. The characteristics of this cell line suggest that it is derived from pre-existing differentiated bile ductal cells from portal tract. However, we cannot rule out the possibility that they derive from committed bile ductal cell precursors that differentiate in vitro. Taken together these results indicate that this cell line may be useful to study duct morphogenesis.

Interestingly, we were only able to isolate cholangiocytes from dissociated acute phase granulomas but not from chronic phase granulomas or dissociated liver fragments. We do not know the reason for this difference but it is tempting to speculate that it reflects intrinsic differences in cellular composition of the granulomas in different phases. For example, while in the acute phase there is intense ductal proliferation with small cells (Fig. 1A–C), during the chronic phase we observe ducts with mucinous generation (hypertrophic cholangiocytes with pathological aspects filled with PAS-positive substance)(Fig. 1D–F). We propose that cells derived from acute phase granulomas have higher viability and proliferative capacity than the ones derived from chronic phase. Regarding the inability to derive cells from liver fragments we believe it may be due to intense connective tissue proliferation that may interfere with epithelial proliferation.

Using the described methodology we were able to isolate epithelial cells from intrahepatic bile ducts only from dissociated acute phase granulomas and not with cultures derived from alternative approaches (whole or dissociated chronic phase granulomas, whole acute phase granulomas; whole or dissociated liver fragments). The reason for this difference is not known. We believe that approaches involving dissociation favors isolation of epithelial cells, since in explants of whole granulomas or liver fragments there is a strong selection for highly migratory cells. This condition favors isolation of connective tissue cells.

To our knowledge this is the first report of a long-term bile duct cell line derived from a schistosomal liver. Also, all other studies dealing with rat or human bile ductular cells report cultures that would go to a limit of six passages or five months of growth. This cell line has been maintained through the fortieth passage and 1 year and eight months of culture without any apparent decrease in proliferation rate. Although cells in the 40^th ^passage are morphologically identical to earlier cultures, we have not performed the extensive characterization done with cells at the 16–18 passages. Also important to the potential use of this cell line to study bile ductular cell physiology is the maintenance of a normal diploid chromosome number. At this time we are unable to say if this cell line will continue indefinitely to proliferate as a continuous cell line but schistosomal livers seem to be a good source of epithelial cells that can be isolated using the method described here.

## Conclusion

The method described here allowed us to isolate long-term cultures of a bile ductal cell line with differentiated characteristics and the capacity for tubulogenesis in a three-dimensional matrix. We believe that this method is a valuable tool to study pathological changes of bile ductules in vitro.

## Abbreviations

BSS: Balanced saline solution; DMEM: Dulbecco's minimal essential medium; FBS: Fetal bovine serum; HEPES: N- [2-Hydroxyethyl] piperazine-N'- [2-ethanesulfonic acid]; IPS: isotonic Percoll solution; PAS: periodic-acid Schiff; PBS: Phosphate buffered saline.

## Competing interests

The author(s) declare that they have no competing interests.

## Authors' contributions

LC established primary cell line cultures, carried out the cell biological studies, participated in designing and interpreting the experiments. RB participated in the design of the study and in the interpretation of the results. CT analyzed the pathology of liver granulomas and participated in discussion and interpretation of the data. AM established primary cultures, conceived of the study, and participated in its design and coordination. All authors read and approved the final manuscript.

## Pre-publication history

The pre-publication history for this paper can be accessed here:
